# Nurses’ perspectives on nurses’ work methods

**DOI:** 10.1590/0034-7167-2023-0374

**Published:** 2024-07-29

**Authors:** João Miguel Almeida Ventura-Silva, Maria Manuela Ferreira Pereira da Silva Martins, Letícia de Lima Trindade, Ana da Conceição Alves Faria, Sónia Cristina da Costa Barros, Mariana Mendes, Ricardo Manuel da Costa Melo, Olga Maria Pimenta Lopes Ribeiro

**Affiliations:** I Universidade do Porto, Instituto de Ciências Biomédicas Abel Salazar. Porto, Portugal; II Cruz Vermelha Portuguesa, Escola Superior de Saúde Norte. Oliveira de Azeméis, Portugal; III CINTESIS@RISE. Porto, Portugal; IV Universidade do Estado de Santa Catarina. Chapecó, Santa Catarina, Brazil; V Centro Hospitalar Universitário São João. Porto, Portugal; VI Universidade Federal de Santa Catarina, Departamento de Enfermagem. Santa Catarina, Brazil; VII Unidade de Investigação em Ciências da Saúde: Enfermagem (UICISA: E). Viseu, Portugal; VIII Escola Superior de Enfermagem do Porto. Porto, Portugal

**Keywords:** Female and Male Nurses, Nursing Care, Work, Patient Safety, Quality Assurance, Health Care, Enfermeros, Atención de Enfermería, Trabajo, Seguridad del Paciente, Garantía de la Calidad de Atención de Salud

## Abstract

**Objectives:**

To analyze nurses’ perspectives on nurses’ work methods in the hospital context.

**Methods:**

A descriptive study with a qualitative approach was conducted in a hospital in northern Portugal, involving 17 nurses. Semi-structured interviews were used for data collection. Data collected between May and June 2023 underwent content analysis, supported by Atlas.ti software.

**Results:**

Three thematic areas emerged: “Nurses’ work methods in a hospital context,” highlighting the conception and components of work methods and the methods in use; “Implementation of nurses’ work methods,” emphasizing influencing factors and challenges to implementation; and “Impact of nurses’ work methods on patients, nurses, and institutions.”

**Final Considerations:**

Nurses’ work methods constitute the structure of nursing care. Some factors influence and some challenges arise in the implementation of these methods, producing impacts on patients, nurses, and institutions.

## RESUMEN

**Objetivos:**

Analizar percepción de enfermeros sobre métodos de trabajo en enfermería en contexto hospitalario.

**Métodos:**

Estudio descriptivo con abordaje cualitativa, realizado en un hospital del norte de Portugal, con participación de 17 enfermeros. Como instrumento de recolecta de datos, recorrido a la entrevista semiestructurada. Datos recolectados entre mayo e junio de 2023, pasaron por análisis de contenido, con suporte del software Atlas.ti.

**Resultados:**

Emergieron tres áreas temáticas: “Métodos de trabajo de enfermeros en contexto hospitalario”, destacándose la concepción y componentes de métodos de trabajo y en uso; “Implementación de métodos de trabajo”, señalándose factores influenciadores y desafíos a la implementación; e “Impacto de métodos de trabajo de enfermeros para los pacientes, enfermeros e instituciones”.

**Consideraciones Finales:**

Los métodos de trabajo constituidos como la estructura de asistencia de enfermería. Hay factores que influencian y desafíos que se quedan a la implementación de esos métodos, produciendo impacto en pacientes, enfermeros e instituciones.

## INTRODUCTION

Since the end of the last century, with the publication of the “To Err is Human” report^(1)^, the importance of establishing a culture of safety in hospitals and ensuring that patients are not harmed by adverse events has been highlighted. The advancement of health technology and patients’ demands regarding health care have brought forth concepts related to the quality of care and patient safety as essential principles and key components of care delivery^(2)^. Quality and safety of care have become a priority, a requirement, and a demand from health organizations aiming to provide sick people with a higher level of health and well-being^(3)^.

International and national organizations, such as the Ordem dos Enfermeiros de Portugal, emphasize the need for nursing practice to be guided by standards that ensure the quality and safety of care using methodologies for organizing nurses’ work^(4)^. Health institutions must define and implement a care delivery method that aligns with the organization’s vision and mission and incorporates all available human and material capital^(5)^.

It is important to highlight that the way nurses organize their work throughout their professional practice is one component linked to the work method. This allows for the assembly of the different ways each nursing professional conceives and implements nursing care to patients in different care contexts^(6)^. It also enables the outlining of the necessary infrastructure to support nursing practice, considering the set of competencies acquired by nurses and the expected outcomes for care^(7)^. However, implementing new methods may require changes in the structure and practices of institutions, a fundamental topic for developing the profession and the qualification of practices.

The educational path of the nurse and the skills acquired throughout their career are aspects that influence the recognition and choice of work methods^(8-9)^. Nursing management also influences the methods adopted by nurses, as it plays an important role in defining practices and promoting the adoption of effective methods^(10)^. The specific characteristics of health institutions also interfere in the configuration of work methods, such as resource availability, organizational culture, and patient profiles^(11)^. Given these aspects, the question arises: What are nurses’ perceptions about the work methods implemented in the hospital context?

## OBJECTIVE

To analyze nurses’ perspectives on nurses’ work methods in the hospital context.

## METHODS

### Ethical Aspects

The study received a favorable opinion from the Ethics Committee and complied with all ethical legal aspects for research involving human beings in Portugal. Participants were informed and clarified about the study’s objectives and purposes and signed informed consent. Confidentiality and anonymity of data were ensured, using the letter N for nurse and SN for specialist nurse followed by a number indicating the order of the interview (N1, N2, SN1, SN2).

### Design and Study Location

This is a descriptive, qualitative study conducted at a central hospital in northern Portugal. The choice of study setting was intentional, as it is a health organization that has developed training related to the methodologies of organizing nursing care, following the Nursing Care Quality Standards (OE 2012) recommended by the Ordem dos Enfermeiros de Portugal. The guidelines of the Consolidated criteria for reporting qualitative research (COREQ) from the Equator Network^(12)^ were followed.

### Population, Inclusion, and Exclusion Criteria

The study participants were defined through non-probabilistic and intentional sampling. Inclusion criteria considered were: being a nurse or specialist nurse (i.e., those professionals who act in direct care) in Portugal; working in the Departments of Medicine, Surgery, and Intensive Medicine and Emergency; and having a service time equal to or greater than one year. Nurses away from work during the study period were excluded. Twenty-one potential participants who met the inclusion criteria were identified, of which 17 agreed to participate in the study, and the rest expressed unavailability.

### Data Collection

Data collection took place between May and June 2023 through interviews using a semi structured guide. The data collection instrument included six closed questions for sociodemographic and professional characterization of participants and six open-ended questions aimed at understanding the concept and components of the work method, facilitating or hindering aspects, and implications that may arise from the implementation of work methods in a hospital context. To assess the comprehension of the questions, the instrument was tested with nurses and specialist nurses working in the Departments of Medicine, Surgery, and Intensive Medicine and Emergency of another institution, i.e., not included in the sampling of this study.

The meeting between the researcher (with the professional category of specialist nurse) and the participants was previously scheduled through a telephone contact provided by the nursing director of the institution where the study was conducted, after authorization from potential participants. Interviews were conducted in a reserved room of the hospital, in the exclusive presence of the participant and the researcher, lasted a minimum of 60 minutes and a maximum of 120 minutes, and were recorded in audio with the participant’s authorization. Given the saturation of data, it was not considered necessary to conduct new interviews or repeat them. The researcher transcribed all interviews in full and sent them by email for participants’ validation.

### Data Analysis

The analysis of the discourses followed the operative proposal of Content Analysis in three phases: pre-analysis (the phase of organizing the data that constituted the research corpus); exploration of the material (the corpus was thoroughly studied to establish the units of registration and context units, from which three thematic axes emerged); and inference and interpretation (the three thematic axes were analyzed based on their constitutive elements by differentiation and by regrouping according to the statements and the study’s objective) (Bardin 2016). Atlas.ti Version 23 software was used, in which interviews were inserted in a Word text file and composed a project. Subsequently, the principal researcher selected the significant excerpts (quotations), to which identifier codes (codes) were attributed, being later organized into three thematic areas (Code groups).

## RESULTS

The majority of participants were women (76.5%), married/in a common-law marriage (82.4%), with an average age of 40.4 years. General care nurses predominated (58.8%) with an average professional experience of 15.2 years. Only 23.5% of the participants had training on the methodology of organizing nursing care.

**Figure 1:**
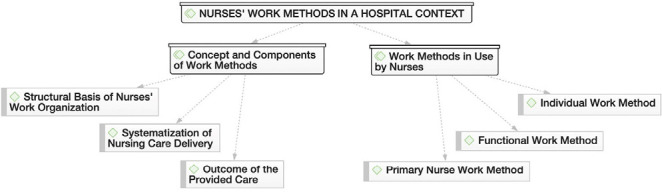
Nurses’ work methods in a hospital contexto. Porto, Portugal, 2023

**Figure 2:**
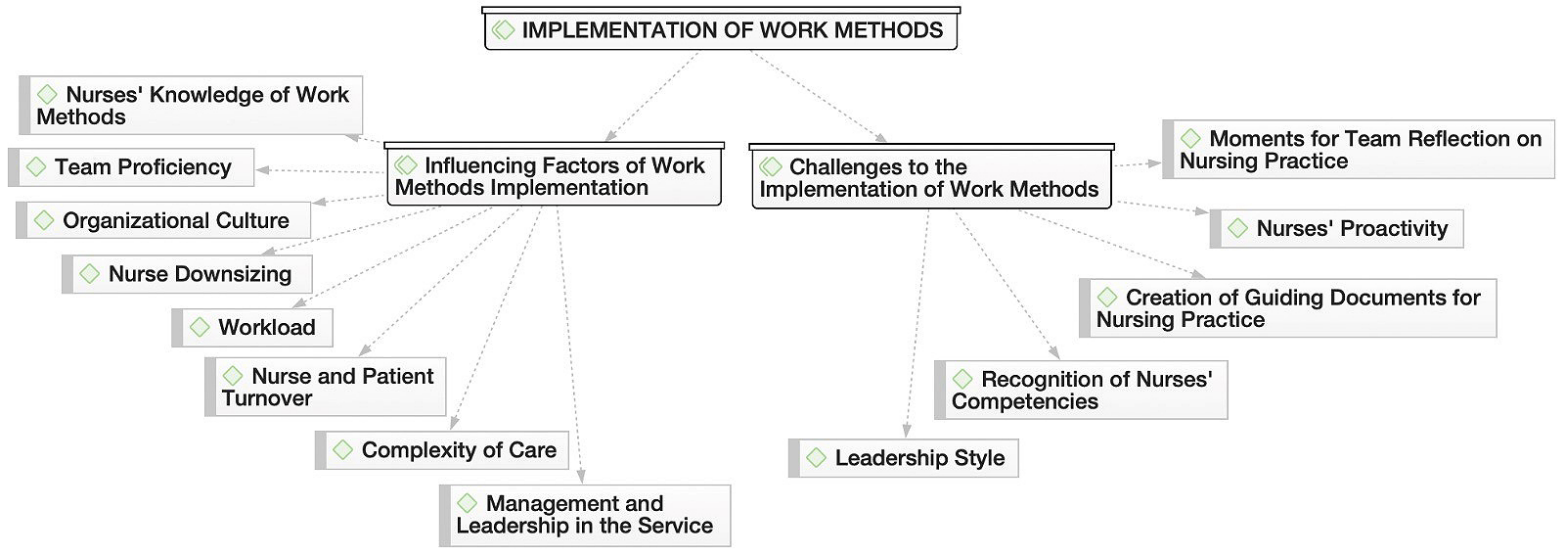
Implementation of work methods, Porto, Portugal, 2023

**Figure 3:**
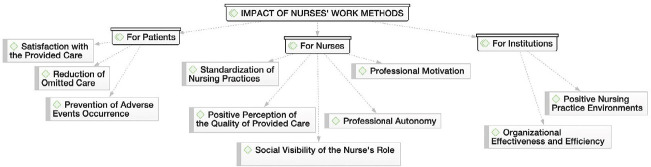
Impact of nurses’ work methods, Porto, Portugal, 2023

From the content of the interviews, three thematic areas emerged, with their respective categories and subcategories (Figures 1, 2, and 3), based on the Nursing Care Quality Standards^(4)^ recommended by the Ordem dos Enfermeiros de Portugal. In the thematic area “Nurses’ work methods in a hospital context,” the discourse of nurses allowed for the identification of two categories: “Conception and components of work methods” and “Work methods in use by nurses,” as illustrated in Figure 1.

In the “Conception and components of work methods” category, participants understand that the work method, particularly its components, forms the structural basis of the organization of nurses’ work, namely the infrastructures that allow for organizing nursing care, considering the expected outcomes of care for the patient:

*It forms the basis of professional existence and the way we organize and apply our knowledge and assist the client in the best way* [...] *with quality and safety.* (N3)

*The work method is extremely important for the team* [...] *it allows us to organize our work and our care, respond to our patients’ needs by prioritizing care* [...] *we can make a supported decision and affirm ourselves among peers and other health professionals, valuing our profession.* (SN6)

The systematic nature of nursing care, supported by nursing theoretical frameworks to meet patients’ needs, was evident in the nurses’ discourse. The organization of care should prioritize a systematic method, with the nurse responsible for its development and documentation.

*The work methods that nurses use aim to organize nursing care* [...] *to provide systematic and targeted responses to the needs of the client requiring our intervention.* (SN1)

*The conceptual basis of our records in the hospital, for example, is framed within Meleis’s transition theory-even the admission notes and initial data collections, the preparation for discharges* [...] *colleagues need to be familiar with what is intended, what the goals and outcomes of our care are.* (N10)

From the participants’ perspective, the outcome of the care provided is directly related to the work method used.

*The method we use allows us to end the shift with the certainty that we have carried out the activities that qualify our contribution to the patients’ recovery.* (N4)

*Only by resorting to a work method is it possible to plan and implement our autonomous and interdependent intervention* [...] *otherwise, it is difficult to accomplish everything.* (N9)

In the “Work methods in use by nurses” category, it was understood these professionals favor the adoption of three of the four traditional work organization methods. Thus, participants highlighted the use of the individual method, the functional method, and the primary nurse method for organizing nursing care.

*The individual method allows us to have a group of patients during the shift, which will imply a more global assistance* [...] *the nurse assumes full responsibility for the delivery of those cares, thus being a more patient-centered delivery.* (N4)

*The task method is used; we know it’s true, even to be quicker in doing things. We use it a lot during night shifts and even on weekends. It helps us because we are working less and the complexity of care remains the same.* (N9)

*In the primary nurse method, studies indicate that there are health gains. This method also promotes professional satisfaction* [...] *it is possible to see that we had a preponderant role.* (SN7)

Regarding the thematic area “Implementation of work methods,” two categories emerged: “Factors influencing the implementation of work methods” and “Challenges to the implementation of work methods,” illustrated in Figure 2.

In the “Factors influencing the implementation of work methods” category, nurses consider knowledge about the philosophy and design of work methods as a facilitating factor for choosing a particular method. By recognizing the principles associated with each work organization methodology, nurses can fully respect them, achieving improvements in patient and professional satisfaction, as well as outcomes for the institution.

*There isn’t a perfect method; everything depends on the circumstances of our context, but if nurses are well-acquainted with work methods, what each one aims for, and the objectives, we can achieve positive results* [...] *there are private hospital entities and specific contexts where the primary nurse is emerging because it yields positive results and nurses are required to know the methodology to guide their performance toward health outcomes.* (N4)

Team proficiency was also a highlighted aspect in the nurses’ discourse, with an emphasis on the varying competence levels of professionals within the teams. Additionally, the downsizing of nurses to teams, including the deficit of professionals and those with little professional experience, could influence the implementation of more efficient work methods:

*Work methods should be suited to the competence stages of the team we have in the service* [...] *the academic and professional training and the professional experience of each member also interfere with the adequacy of methods.* (SN5)

*The adoption of a work method is related to the number of nurses and the nurses who are on the shift* [...] *we have a complex team, we have newly graduated colleagues, colleagues with vast professional experience* [...] *the number of nurses and the nurses who are on the shift are important aspects.* (SN3)

Another factor highlighted refers to the workload, nurse and patient turnover, and complexity of care. For participants, the heavy workload, high turnover of nurses, as well as patients’ level of dependence and their length of stay in the services influence work methods.

*There are difficulties in executing our work method because we have a high turnover of patients who stay for a short period. Another aspect that makes it difficult for us is related to the heavy workload we have in a shift, which is quite increased; human resources are few.* (N10)

*The workload and turnover are very high in hospitalization, and the pressure of being with less adequate ratios makes us leave something undone that could be relevant and is not covered due to lack of time and sometimes due to difficulty in managing the shift activities.* (SN7)

*Unfortunately, resources are increasingly scarce, and healthcare demands are more complex with an aging population* [...] *needs are increasingly greater. The choice of patient-centered methods is more beneficial to respond to this population and allows for a more substantiated decision-making.* (N5)

As an added value for implementing the work method and team development, nurses also identified management and leadership in the service, as well as an organizational culture that values nursing work and patient centrality.

*A neutral leadership is not a leadership that enhances decision-making, it is not a leadership that enhances progress* [...] *nor does it value work methods.* (SN2)

*People in management positions should understand which method values the work of nurses and what nurses do.* (SN7)

*The adoption of a particular work methodology depends on the organizational culture and on the reality of each service and institution. It is an added value that can promote the quality and safety of nursing care.* (SN5)

In the category “Challenges to the implementation of work methods,” it was possible to identify a set of aspects that may compromise the implementation of more suitable work methods for the needs of patients, professionals, and institutions. Nurses acknowledge that the lack of moments for reflection on practice makes it difficult to organize and perform their work.

*Reflection on practice would help us to better organize the care to be provided. Discussion about adverse events that occur or team difficulties is extremely important for all of us to grow.* (SN2)

*Debriefings also in more complex situations, emergency situations, differentiated intervention situations that we are still not accustomed to should also be contemplated for the professional development of the team and for how nurses can organize their work.* (N2)

On the other hand, they noted the need to create guiding documents for clinical practice that enable the standardization of nursing care. It is believed that directing the nurse’s work and how it is executed contributes to more positive health outcomes and aligns with the recommended work method.

*For example, in the patient’s discharge letter, there must be a standard so that everyone fills it out in the same way* [...] *involving nurses in this process.* (N3)

*It’s important to achieve health outcomes. We can only achieve this if there is a standard, a standardized way that allows activities to be carried out in the same way and thus we can ensure that there is no deviation from the recommended work method.* (SN2)

Participants also highlighted that valuing the competencies developed by nurses and investments in the professional training process are challenges to the implementation of work methods. From this perspective, valuing the proactivity of these professionals has the potential to promote autonomy and evidence-based decision-making, as well as to broaden the social visibility of the nurse’s role.

*More and more, it is important within the team that there is an appreciation of professionals individually and that the professional competencies that each one acquires are recognized. If in the team I am an expert in a certain area, I have an added competence, it is good that it is taken advantage of.* (N2)

*When our peers recognize our value, our competencies, it is gratifying: in the end, we feel valued. Undoubtedly, this issue is an aspect that is increasingly posed in the organization of nurses’ work.* (SN6)

*In our contexts, each nurse must have a proactive attitude, in the sense of looking for work methods that meet the enhancement of decision-making and professional autonomy.* (N5)

The leadership style adopted in the service can also be challenging to the implementation of work methods. For participants, transformational leadership presents itself as a viable alternative, considering it stimulates more positive practice environments.

*Nurses are professionals who seek out and stimulate each other, who are always undergoing training independently and unpaid, making personal investments, and often end up being restrained by leadership.* (N8)

*Leadership is also essential* [...] *the leadership style should encourage us* [...] *we manage to achieve professional satisfaction, develop our work with satisfaction, and promote a healthy work environment.* (SN6)

In the thematic area “Impact of nurses’ work methods,” three categories were identified: “For patients,” “For nurses,” and “For institutions,” as illustrated in Figure 3.

According to the participants, the impact of nurses’ work methods “for patients” is seen in safer, quality health care, with repercussions on patient satisfaction with the provided care.

*Nurses’ work methods value and promote safety. It’s not doing for the sake of doing but doing sustainably* [...] *responding to the needs of the users, leading to satisfaction with the care.* (SN6)

*The methodology adopted in each service seeks to respond to pillars such as the quality and safety of care but also to worry about patient satisfaction because if the patients are satisfied, it means that the professionals have done a good job.* (N1)

The reduction of omitted care and the prevention of adverse events are related to the implementation of more efficient work methods, focused on the patient and committed to the safety of nursing care.

*The needs of the patient sometimes end up not being as satisfied as they should be, which is related to various factors. Not doing or doing too late is one of the major problems that are not always discussed and try to be addressed by the colleague who comes next.* (SN4)

*The way we assist patients can promote or hinder adverse events. The fact of being more or less involved with the patient influences our planning and implementation of care.* (SN7)

For the participants, the work methods also present positive impacts “for nurses” as they provide standardization of care practices and a positive perception of the quality of care provided, potentially enhancing the social visibility of the nurse’s role in various work contexts.

*The method* [...] *is important to standardize and provide better care to patients.* (N6)

*We also have our expectations about our workplace* [...] *we aim to provide quality care, to do our job better.* (SN1)

*The work methodology that nurses adopt will give us visibility* [...] *ensuring response to the best care with quality and safety.* (SN5)

Moreover, implementing appropriate work methods has repercussions on autonomy and professional satisfaction, as well as on the structure and organization of care.

*There are work methods that are enhancers of decision-making and autonomy* [...] *and others that are frankly more restricting* [...] *it should be allowed for the nurse to actively participate in the planning, execution, and evaluation of the provided care.* (N7)

*The way we provide and organize nursing care will influence not only the client’s satisfaction but also the professionals’ satisfaction in their workplace, potentially influencing the outcome indicators and what we can achieve from our practice.* (N8)

*The adoption of a method has a significant influence on professionals’ satisfaction. If professionals are not satisfied and are not directed, our performance may be compromised, reflecting dissatisfaction.* (SN5)

In this thematic area, the testimonials of nurses also show positive effects “for institutions,” specifically in organizational effectiveness and efficiency, promoting more positive practice environments for nursing.

*If the institution is directed toward quality indicators and if the nurse works toward these indicators, the way he organizes his work will be reflected in increased gains, satisfaction, safety, and quality.* (SN4)

*There are work methods more focused on care-sensitive indicators* [...] *those that aim at patient centrality and thus promote good organizational performance.* (SN5)

*The method influences positive environments as well as the opposite* [...] *what is often lacking for the team is the so-called emotional salary* [...] *managers cannot give a monetary reward* [...] *there are recognitions/attitudes that help.* (N7)

## DISCUSSION

Regardless of where nursing care is provided to patients, the existence of a work method adopted by the nurse is fundamental to achieving outcomes and projecting the social visibility of this professional role. Since the beginning of the professionalization of nursing, issues related to the nurse’s work have been discussed. Imogene King already warned about the need for nurses to organize their work. The author highlighted that the way each professional cares for patients and the existence of a care documentation system would allow nursing to be distinguished from other health professions, particularly through the identification of the real needs of the patient and family caregivers, as well as through the implementation, evaluation, and continuity of care^(14-15)^.

In the hospital context, work methods constitute the structure of the nurse’s work, that is, the way each nurse, in their professional performance, responds to what is the social mandate of the nursing profession. It is understood that how each nursing professional identifies the needs of patients, formulates diagnoses, defines and implements interventions, as well as evaluates the results of these interventions, allows structuring the nurse’s work. A work method enables the organization and nursing care delivery to patients; it is guided by values and beliefs and is described as the independent or collaborative approach of nurses in the direct care of a group of patients^(10,16)^. An important point to highlight is that the conception and organization of the nurse’s work are based on management theories and essentially on nursing theories^(10)^-data corroborated in this investigation.

It was found that the work method is central to defining interdependent and autonomous nursing interventions. Not only does it involve a list of activities to be performed, but it also focuses on how nurses choose what they want to do, with an orientation toward the real needs of the patient and family^(17)^, emphasizing on autonomous nursing interventions. It was identified that the work methods most adopted by nurses in the hospital context were the individual, functional, and primary nurse methods, all focused on patient centrality, with positive implications for satisfaction with the care provided. It is understood that the methodologies for organizing the nurse’s work should focus on the development of care, attending to its complexities, and that patient care should develop according to the care needs and responsibilities of each professional in the care environment^(18)^.

A justification for the team-based work method not being mentioned by nurses as a work method in use was the realization of the study in departments where the organization of nursing care is centered on the person. Moreover, in this organization, assigning patients to nurses primarily considers continuity of care, which, whenever possible, should be provided by the same nurse.

Some facilitators and obstacles to the implementation of work methods were found: the scarcity of nurses; the downsizing of professionals in services and the number of patients assigned to each nurse; the characteristics of practice environments, particularly the organizational culture; the knowledge held by nurses, reflecting on autonomy in decision-making; and teamwork. Some authors point out that reflection on practice, the recognition of nurses’ competencies, and leadership style are challenges to the implementation of nursing care organization methodologies^(5,11,19)^.

It is highlighted that, in addition to what is mentioned in the literature, it was observed that the complexity of care, the workload, the turnover of nurses and patients in services, as well as management and leadership in the service, can influence the implementation of work methods. Research indicates that the leadership advocated by the nurse manager plays a decisive role in the quality of the care process and the team because the way a nurse provides care and the competencies inherent to the manager tend to enhance the quality of the service provided to patients and families^(6,20-22)^.

Regarding other challenges identified, nurses signal the importance of having guiding documents for clinical practice that allow for the standardization of care delivery. On the other hand, valuing professional competencies and the proactivity of nurses are shown to be crucial for implementing work methods aimed at satisfying patients and professionals, and ensuring quality and safety in nursing care.

It was also perceived that the way nurses organize their work and provide care to patients and families affects the patients, nurses, and institutions. Literature reinforces that promoting patient satisfaction, reducing adverse events and omitted care, and ensuring safe care represent significant gains^(6,16)^.

Moreover, it is important to emphasize that nursing care delivery, based on an organizational methodology, impacts the institution by reducing healthcare costs while simultaneously achieving success with the health care provided to patients in a context of increasing healthcare expenses, reforms, expectations, and consumer feedback^(14,20,23)^. It was evidenced that nurses’ work methods influence nursing practice environments, which are expected to be positive, fostering adequate working conditions, satisfaction with the care provided, professional satisfaction, and greater involvement of professionals with institutional policies and strategies-findings in line with the literature^(24-26)^.

Reflecting on the extensive data related to the organization of nurses’ work^(27)^, it becomes essential to consider that given the discussed aspects, there is not just one single work method capable of meeting the needs of patients and families but rather a hybridization of them according to the factors that cause this need. It becomes imperative to consider these factors, namely the beliefs, values, and perceptions of nurses about their professional practice, as well as other aspects related to care contexts and health organizations, to more effectively ensure health safety and quality.

### Study Limitations

As for the limitations, it is highlighted that the study was conducted only in some departments of a hospital institution in the country, which may not fully reflect the reality of other hospitals in Portugal. Additionally, the number of participants could also be considered a limitation to the extent that it may have compromised the identification of other perspectives regarding the topic under study.

### Contributions to the field of Nursing

Considering the demands related to the quality of health care, this study contributes to issues related to the organization of nurses’ work in terms of the flexibility of nurses to appropriate the concept of hybridization of work methods aligned with organizational culture. The findings are important for nursing management and promoting quality and safety in care.

## FINAL CONSIDERATIONS

The organization of nursing care, based on work methods, is presented as a necessary premise for the quality and safety of care. It is considered that a work method constitutes the structure of the nurses’ work, which allows them to systematize the identification of needs, definition of problems, implementation of nursing interventions, and evaluation of the care provided.

It is emphasized that the promoting factors influence the adoption of work methods more directed to person-centered care; however, the presence of challenges reflects on the work organization methodologies, with potential impact on patients, nurses, and institutions, affecting the quality of assistance. For this, it is suggested to study the work methods of nurses that best respond to the demands of each care context, with systematic dialogue and monitoring of advances for the quality of nursing care. It is also suggested to conduct studies related to this theme but aimed at nurse managers, as these professionals have significant potential to contribute to the management of nurses’ work in favor of safe and quality care for patients.
