# Supplementary material for: Nurses’ perspectives on nurses’ work methods
Source: Rev Bras Enferm. 2024 Jul 29;77(3):e20230374. doi: 10.1590/0034-7167-2023-0374 (PMC11290740; doi:10.1590/0034-7167-2023-0374)
Supplement: Translation

# Translation: Olhar dos enfermeiros sobre os métodos de trabalho em enfermagem

**DOI:** 10.1590/0034-7167-2023-0374pt

**Authors:** João Miguel Almeida Ventura-Silva, Maria Manuela Ferreira Pereira da Silva Martins, Letícia de Lima Trindade, Ana da Conceição Alves Faria, Sónia Cristina da Costa Barros, Mariana Mendes, Ricardo Manuel da Costa Melo, Olga Maria Pimenta Lopes Ribeiro

**Affiliations:** I Universidade do Porto, Instituto de Ciências Biomédicas Abel Salazar. Porto, Portugal; II Cruz Vermelha Portuguesa, Escola Superior de Saúde Norte. Oliveira de Azeméis, Portugal; III CINTESIS@RISE. Porto, Portugal; IV Universidade do Estado de Santa Catarina. Chapecó, Santa Catarina, Brasil.; V Centro Hospitalar Universitário São João. Porto, Portugal; VI Universidade Federal de Santa Catarina, Departamento de Enfermagem. Santa Catarina, Brasil; VII Unidade de Investigação em Ciências da Saúde: Enfermagem (UICISA: E). Viseu, Portugal; VIII Escola Superior de Enfermagem do Porto. Porto, Portugal

**Keywords:** Enfermeiros, Cuidados de Enfermagem, Trabalho, Segurança do Paciente, Garantia da Qualidade dos Cuidados de Saúde

## Abstract

**Objetivos:**

Analisar a percepção dos enfermeiros sobre os métodos de trabalho em enfermagem no contexto hospitalar.

**Métodos:**

Estudo descritivo com abordagem qualitativa, realizado num hospital do norte de Portugal, com participação de 17 enfermeiros. Como instrumento de coleta de dados, recorreu se à entrevista semiestruturada. Os dados, recolhidos entre maio e junho de 2023, passaram por análise de conteúdo, com suporte do software Atlas.ti.

**Resultados:**

Emergiram três áreas temáticas: “Métodos de trabalho dos enfermeiros em contexto hospitalar”, destacando-se a concepção e componentes dos métodos de trabalho e os métodos em uso; “Implementação dos métodos de trabalho”, salientando-se fatores influenciadores e desafios à implementação; e “Impacto dos métodos de trabalho dos enfermeiros para os pacientes, enfermeiros e instituições”.

**Considerações Finais:**

Os métodos de trabalho constituem-se como a estrutura da assistência de enfermagem. Existem fatores que influenciam e desafios que se colocam à implementação desses métodos, produzindo impacto nos pacientes, enfermeiros e instituições.

## INTRODUÇÃO

Desde o final do século passado, pela divulgação do Relatório “*To Err is Human*”, realçou-se a importância de estabelecer uma cultura de segurança nos hospitais e garantir que os pacientes não fossem prejudicados por eventos adversos^(1)^. O avanço da tecnologia em saúde e as exigências dos pacientes no tocante à assistência em saúde fazem emergir conceitos relacionados com a qualidade dos cuidados e a segurança do paciente, como sendo princípios essenciais e componentes-chave da prestação de cuidados^(2)^. A qualidade e segurança dos cuidados passa a ser uma prioridade, um requisito e uma exigência das organizações de saúde, visando proporcionar às pessoas doentes um maior nível de saúde e bem-estar^(3)^.

Organizações internacionais e nacionais, como a Ordem dos Enfermeiros de Portugal, salientam a necessidade de a prática de enfermagem ser orientada por padrões que garantam a qualidade e segurança dos cuidados, com uso de metodologias de organização do trabalho do enfermeiro^(4)^. As instituições de saúde devem definir e implementar um método de prestação de cuidados, que se alinhe com a visão e missão da organização e incorpore todo o capital humano e material disponível^(5)^.

Importa destacar que a forma como os enfermeiros organizam o trabalho ao longo do seu exercício profissional constitui-se como um dos componentes ligados ao método de trabalho. Este permite reunir as diferentes formas pelas quais cada profissional de enfermagem concebe e implementa os cuidados de enfermagem aos pacientes nos diferentes contextos de cuidados^(6)^. Também possibilita delinear as infraestruturas necessárias à sustentação da prática de enfermagem, considerando o conjunto de competências adquiridas pelos enfermeiros e os resultados esperados para a assistência^(7)^. Entretanto, a implementação de novos métodos pode exigir mudanças na estrutura e nas práticas das instituições, que é um tema fundamental para o desenvolvimento da profissão e qualificação das práticas.

O percurso formativo do enfermeiro e as habilidades adquiridas ao longo da carreira são aspectos que influenciam o reconhecimento e escolha dos métodos de trabalho^(8-9)^. A gestão em enfermagem também tem influência nos métodos adotados pelos enfermeiros, pois desempenha papel importante na definição das práticas e na promoção da adoção de métodos eficazes^(10)^. As características específicas das instituições de saúde também interferem na configuração dos métodos de trabalho, como a disponibilidade de recursos, a cultura organizacional e o perfil dos pacientes^(11)^. Diante desses aspectos, questiona-se: Quais as percepções dos enfermeiros acerca dos métodos de trabalho implementados no contexto hospitalar?

## OBJETIVO

Analisar a percepção dos enfermeiros sobre os métodos de trabalho em enfermagem em contexto hospitalar.

## MÉTODOS

### Aspectos éticos

O estudo obteve parecer favorável do Comitê de Ética e atendeu a todos os aspectos ético-legais para pesquisas que envolvam seres humanos em Portugal. Os participantes foram informados e esclarecidos sobre os objetivos e finalidades do estudo e assinaram o consentimento informado. Garantiu se a confidencialidade e o anonimato dos dados, utilizando a letra E para enfermeiro e EE para enfermeiro especialista, seguida de número indicando a ordem da entrevista (E1, E2, EE1, EE2).

### Desenho e local do estudo

Estudo descritivo, de natureza qualitativa, realizado em um hospital central do norte de Portugal. A escolha do cenário do estudo foi intencional, por se tratar de uma organização de saúde que tem desenvolvido formação relativa às metodologias de organização dos cuidados de enfermagem aos enfermeiros, seguindo os Padrões de Qualidade dos Cuidados de Enfermagem (OE, 2012), preconizados pela Ordem dos Enfermeiros de Portugal.

Seguiram-se as diretrizes do *Consolidated criteria for reporting qualitative research* (COREQ), da Rede Equator^(12)^.

### População, critérios de inclusão e exclusão

A definição dos participantes do estudo foi realizada por amostragem não probabilística e intencional. Como critérios de inclusão, considerou-se: ser enfermeiro ou enfermeiro especialista (ou seja, aqueles profissionais que, em Portugal, atuam no cuidado direto); atuar nos Departamentos de Medicina, Cirurgia e Medicina Intensiva e Urgência; e possuir tempo de serviço superior ou igual a um ano. Excluíram-se os enfermeiros afastados do trabalho durante o período do estudo. Foram identificados 21 potenciais participantes que cumpriam os critérios de inclusão, sendo que 17 aceitaram participar do estudo, e os demais manifestaram indisponibilidade.

### Coleta dos dados

A coleta de dados decorreu entre maio e junho de 2023, mediante entrevista e utilização de um roteiro semiestruturado. O instrumento de coleta de dados contava com seis questões fechadas para caracterização sociodemográfica e profissional dos participantes e seis questões abertas direcionadas à compreensão do conceito e componentes do método de trabalho, aspectos facilitadores ou dificultadores e implicações que possam surgir da implementação dos métodos de trabalho em contexto hospitalar. Para avaliar a compreensão das questões, o instrumento foi testado com enfermeiros e enfermeiros especialistas, que atuavam em Departamentos de Medicina, Cirurgia e Medicina Intensiva e Urgência de outra instituição, ou seja, não contemplados na amostragem deste estudo.

O encontro entre o pesquisador (com categoria profissional de enfermeiro especialista) e os participantes foi previamente agendado mediante contato telefônico, fornecido pelo enfermeiro diretor da instituição onde decorreu o estudo, após autorização dos potenciais participantes. As entrevistas foram realizadas em uma sala reservada do hospital, na presença exclusiva do participante e do pesquisador; tiveram duração mínima de 60 minutos e máxima de 120 minutos; e foram registradas por meio de gravação de áudio, com autorização do participante. Dada a saturação dos dados, não se considerou necessária a realizar novas entrevistas e/ou repeti-las. O pesquisador transcreveu todas as entrevistas integralmente e as enviou por correio eletrônico para validação dos participantes.

### Análise dos dados

A análise dos discursos seguiu a proposta operativa da Análise de Conteúdo, em três fases: pré análise (fase de organização dos dados que constituíram o corpus da pesquisa); exploração do material (o corpus foi estudado profundamente, com o objetivo de estabelecer as unidades de registro e unidades de contexto, emergindo três eixos temáticos); e inferência e interpretação (os três eixos temáticos foram analisados com base nos seus elementos constitutivos, por diferenciação e por reagrupamento segundo as falas e o objetivo do estudo) (Bardin, 2016). Foi usado o software Atlas.ti Versão 23, no qual as entrevistas foram inseridas em arquivo de texto Word e compuseram um *project*. Na sequência, o pesquisador principal selecionou os trechos significativos (*quotations*), aos quais foram atribuídos códigos identificadores (*codes*), sendo posteriormente organizados em três áreas temáticas (*Code groups*).

## RESULTADOS

A maioria dos participantes eram mulheres (76,5%), casadas/em união estável (82,4%) e com uma média de idade de 40,4 anos. Predominaram os enfermeiros de cuidados gerais (58,8%), com tempo médio de experiência profissional de 15,2 anos. Apenas 23,5% dos participantes tiveram formação sobre metodologia de organização dos cuidados de enfermagem.

Do conteúdo das entrevistas, emergiram três áreas temáticas, com suas respectivas categorias e subcategorias (Figuras 1, 2 e 3), baseando-se nos Padrões de Qualidade dos Cuidados de Enfermagem^(4)^, da Ordem dos Enfermeiros de Portugal.

**Figura 1:** Métodos de trabalho dos enfermeiros em contexto hospitalar. Porto, Portugal, 2023

**Figura 2:** Implementação dos métodos de trabalho, Porto, Portugal, 2023

**Figura 3:** Impacto dos métodos de trabalho dos enfermeiros, Porto, Portugal, 2023

Na área temática “Métodos de trabalho dos enfermeiros em contexto hospitalar”, o discurso dos enfermeiros permitiu identificar duas categorias “Concepção e componentes dos métodos de trabalho” e “Métodos de trabalho em uso pelos enfermeiros”, conforme ilustra a Figura 1.

Na categoria “Concepção e componentes dos métodos de trabalho”, os participantes entendem que o método de trabalho, em particular os seus componentes, são a base estrutural da organização do trabalho do enfermeiro, nomeadamente as infraestruturas que permitem a organização da assistência de enfermagem, considerando os resultados esperados da prestação de cuidados para o paciente:

*É base da existência profissional e a forma como nós organizamos e aplicamos os nossos conhecimentos e ajudamos o cliente da melhor forma* [...] *com qualidade e segurança.* (E3)

*O método de trabalho é extremamente importante para a equipa* [...] *permite organizar o nosso trabalho e os nossos cuidados, responder às necessidades dos nossos doentes priorizando os cuidados* [...] *conseguimos uma tomada de decisão sustentada e afirmarmo-nos entre os pares e os outros profissionais de saúde, valorizando a nossa profissão.* (EE6)

A sistematização da assistência de enfermagem, sustentada nos referenciais teóricos de enfermagem, para responder às necessidades dos pacientes, ficou evidente no discurso dos enfermeiros. A forma como é feita a organização do cuidado deve privilegiar um método sistemático, sendo o enfermeiro o responsável pelo seu desenvolvimento e documentação.

*Os métodos de trabalho que os enfermeiros usam têm o intuito de organizar os cuidados de enfermagem* [...] *dar respostas sistemáticas e direcionadas às necessidades do cliente que necessita da nossa intervenção.* (EE1)

*A base conceptual dos nossos registos no hospital, por exemplo, está enquadrada na teoria de transições da Meleis - mesmo as notas de admissão e as coletas de dados iniciais, a preparação para as altas* [...] *os colegas têm que estar familiarizados com o que se pretende, quais são os objetivos e os resultados dos nossos cuidados.* (E10)

Na perspectiva dos participantes, o produto dos cuidados prestados está diretamente relacionado com o método de trabalho usado.

*O método que usamos permite-nos terminar o turno com a certeza que realizamos as atividades que qualificam a nossa contribuição para a recuperação dos doentes.* (E4)

*Só com recurso a um método de trabalho é que se torna possível planear e implementar a nossa intervenção autónoma e interdependente* [...] *caso contrário, é difícil ter tempo para concretizar tudo.* (E9)

Na categoria “Métodos de trabalho em uso pelos enfermeiros”, percebeu-se que esses profissionais privilegiam a adoção de três dos quatro métodos tradicionais de organização do trabalho. Assim, os participantes destacaram a utilização do método individual, do método funcional e do método por enfermeiro de referência, para a organização da assistência de enfermagem.

*O método individual permite que tenhamos um grupo de doentes no turno, o que implicará uma assistência mais global* [...] *o enfermeiro assume total responsabilidade pela prestação desses cuidados, sendo portanto uma prestação mais centrada no doente.* (E4)

*O método à tarefa é usado, sabemos que é verdade, até para sermos mais rápidos a fazer as coisas. Usamos muito nos turnos da noite e até aos fins de semana. Isso ajuda-nos porque estamos menos a trabalhar e a complexidade de cuidados mantém-se a mesma.* (E9)

*No método de enfermeiro de referência, os estudos apontam que existe ganhos em saúde. Este método promove também a satisfação profissional* [...] *consegue-se perceber que tivemos um papel preponderante.* (EE7)

Em relação à área temática “Implementação dos métodos de trabalho”, emergiram duas categorias: “Fatores influenciadores da implementação dos métodos de trabalho” e “Desafios à implementação dos métodos de trabalho”, ilustradas na Figura 2.

Na categoria “Fatores influenciadores da implementação dos métodos de trabalho”, os enfermeiros consideram que ter conhecimento sobre a filosofia e concepção dos métodos de trabalho era um fator facilitador da opção por determinado método. Ao reconhecer os princípios associados a cada metodologia de organização do trabalho, os enfermeiros podem respeitá-los na sua integridade, obtendo melhorias na satisfação dos pacientes e dos profissionais, bem como nos resultados para a instituição.

*Não há um método perfeito, tudo depende das circunstâncias do nosso contexto, mas se os enfermeiros conhecerem bem os métodos de trabalho, o que pretende cada um, os objetivos, conseguimos obter resultados positivos* [...] *há entidades hospitalares privadas e em contextos específicos, em que está a surgir o enfermeiro de referência, porque permite resultados positivos, e os enfermeiros são obrigados a conhecer a metodologia, para orientar o seu desempenho para os resultados em saúde.* (E4)

A proficiência da equipe também foi um aspecto ressaltado no discurso dos enfermeiros, com ênfase nos diferentes níveis de competência dos profissionais que compõem as equipes. Soma-se a isso ao dimensionamento de enfermeiros nas equipes, incluindo o déficit de profissionais e os que têm pouca experiência profissional, que poderá constituir-se como um fator influenciador da implementação dos métodos de trabalho mais eficientes:

*Os métodos de trabalho devem ser adequados aos estádios de competências da equipa que nós temos no serviço* [...] *a formação académica e profissional e a experiência profissional de cada elemento também interferem com a adequação dos métodos.* (EE5)

*A adoção do método de trabalho relaciona-se com o número de enfermeiros e com os enfermeiros que estão no turno* [...] *temos uma equipa complexa, temos gente recém-formada, colegas já com uma vasta experiência profissional* [...] *o número de enfermeiros e os enfermeiros que estão no turno são aspetos importantes.* (EE3)

Outro fator destacado refere-se à carga de trabalho, ao *turnover* de enfermeiros e pacientes e complexidade dos cuidados. Para os participantes, a sobrecarga de atividades, a rotatividade dos enfermeiros, bem como o nível de dependência e o tempo de permanência dos pacientes nos serviços acabam por influenciar os métodos de trabalho.

*Existem dificuldades na execução do nosso método de trabalho, porque temos uma grande rotação de doentes, que estão pouco tempo internados. Também outro aspeto que nos dificulta prende-se com a grande carga de trabalho que temos num turno, que é bastante acrescida; os recursos humanos são poucos.* (E10)

*A sobrecarga de trabalho e o* turnover *é muito aumentado no internamento, a pressão de estarmos com rácios menos adequados faz com que se deixe alguma coisa por fazer que poderia ser relevante e não é contemplada pela falta de tempo e às vezes por dificuldade em gerir as atividades do turno.* (EE7)

*Infelizmente os recursos são cada vez menos e os cuidados de saúde mais exigentes e complexos, com uma população mais envelhecida* [...] *as necessidades são cada vez mais. A opção por métodos mais centrados no cliente são mais benéficos para responder a esta população e permitir uma tomada de decisão mais fundamentada.* (E5)

Como uma mais-valia para a implementação do método de trabalho e desenvolvimento da equipe, os enfermeiros também identificaram a gestão e a liderança no serviço, assim como uma cultura organizacional que valoriza o trabalho do enfermeiro e a centralidade no paciente.

*Uma liderança neutra não é uma liderança que potencia as tomadas de decisão, não é uma liderança que potencia o progresso* [...] *nem valoriza os métodos de trabalho.* (EE2)

*As pessoas que estão em posições de gestão deveriam perceber qual o método que valoriza o trabalho dos enfermeiros e aquilo que os enfermeiros fazem.* (EE7)

*A adoção de uma determinada metodologia de trabalho depende da cultura organizacional e também da realidade de cada serviço e de cada instituição. É uma mais-valia que pode promover a qualidade e segurança dos cuidados do enfermeiro.* (EE5)

Na categoria “Desafios à implementação dos métodos de trabalho”, foi possível determinar um conjunto de aspectos que pode comprometer a implementação de métodos de trabalho mais adequados às necessidades dos pacientes, profissionais e instituições. Os enfermeiros reconhecem que a falta de momentos para reflexão sobre a prática dificulta a organização e realização do seu trabalho.

*A reflexão sobre a prática ajudava-nos a organizar melhor os cuidados a prestar. A discussão sobre os eventos adversos que ocorrem ou as dificuldades de equipa é extremamente importante para todos crescermos.* (EE2)

*Os* debriefings *também nas situações mais complexas, as situações de emergência, as situações de intervenção diferenciadas que a gente ainda não esteja habituado também devem ser contemplados para o desenvolvimento profissional da equipa e para a forma como os enfermeiros poderão organizar o seu trabalho.* (E2)

Por outro lado, notaram a necessidade da criação de documentos orientadores da prática assistencial e que viabilizem a padronização da assistência de enfermagem. Acredita-se que direcionar o trabalho do enfermeiro e a forma como é executado contribui para resultados em saúde mais positivos e alinhados com o método de trabalho preconizado.

*Por exemplo, na carta de alta do doente, é importante que haja uma norma, para que todos preencham da mesma forma* [...] *envolver os enfermeiros nesse processo.* (E3)

*É importante a obtenção de resultados em saúde. Só o conseguimos se existir um padrão, uma forma padronizada que permita a realização das atividades da mesma forma e assim conseguimos que não haja enviesamento do método de trabalho preconizado.* (EE2)

Os participantes também destacaram que a valorização das competências desenvolvidas pelos enfermeiros e os investimentos no processo formativo profissional são desafios à implementação dos métodos de trabalho. Sob tal perspectiva, valorizar a proatividade desses profissionais tem potencial para promover a autonomia e a tomada de decisão sustentada, além de ampliar a visibilidade social do papel do enfermeiro.

*Cada vez mais é importante dentro da equipa que haja uma valorização dos profissionais de forma individual, que sejam reconhecidas as competências profissionais que cada um adquire. Se na equipa eu sou expert em determinada área, tenho uma competência acrescida, é bom que seja aproveitada.* (E2)

*Quando os nossos pares reconhecem o nosso valor, as nossas competências são gratificantes: no fundo, sentimo-nos valorizados. Sem dúvida que esta questão é um aspeto que se coloca cada vez mais na organização do trabalho do enfermeiro*. (EE6)

*Nos nossos contextos, cada enfermeiro deve ter uma atitude proactiva, no sentido de procurarmos métodos de trabalho que vão ao encontro da potenciação da tomada de decisão e da autonomia profissional.* (E5)

O estilo de liderança adotado no serviço também pode ser desafiador à implementação dos métodos de trabalho. Para os participantes, a liderança transformacional apresenta-se como alternativa viável, considerando que estimula ambientes de prática mais positivos.

*Os enfermeiros são profissionais que procuram, que se estimulam, que estão sempre a fazer formação independente e não remunerada, fazem investimento pessoal e muitas vezes acabam por ser castrados pela liderança.* (E8)

*A liderança também é essencial* [...] *o estilo de liderança deve-nos incentivar* [...] *conseguimos atingir a satisfação profissional, a desenvolvermos o nosso trabalho com satisfação e potenciar um ambiente de trabalho saudável.* (EE6)

Na área temática “Impacto dos métodos de trabalho dos enfermeiros”, identificaram-se três categorias: “Para os pacientes”, “Para os enfermeiros” e “Para as instituições”, ilustradas na Figura 3.

Segundo os participantes, o impacto dos métodos de trabalho “para os pacientes” é visto em uma assistência à saúde mais segura, de qualidade, com repercussões na satisfação dos pacientes com os cuidados prestados.

*Os métodos de trabalho valorizam e promovem a segurança. Não é um fazer por fazer, mas um fazer sustentado* [...] *dar resposta às necessidades dos utentes, levando à satisfação com os cuidados.* (EE6)

*A metodologia adotada em cada serviço procura dar resposta a pilares como a qualidade e segurança dos cuidados, mas também preocupar-se com a satisfação dos doentes, isto porque, se os doentes ficam satisfeitos, significa que os profissionais fizeram um bom trabalho.* (E1)

A diminuição de cuidados omissos e a prevenção da ocorrência de eventos adversos estão relacionadas com a implementação de métodos de trabalho mais eficientes, centrados no paciente e comprometidos com a segurança da assistência de enfermagem.

*As necessidades do doente, por vezes, acabam por não ser satisfeitas como deveriam ser, o que está relacionado com diversos fatores. O não fazer ou fazer tardiamente é um dos grandes problemas que nem sempre são discutidos e tenta-se colmatar com o colega que vem a seguir.* (EE4)

*A forma como assistimos os doentes poderá ser promotora ou dificultadora de eventos adversos. O facto de nos envolvermos mais ou menos com o doente influencia o nosso planeamento e implementação dos cuidados.* (EE7)

Para os participantes, os métodos de trabalho também apresentam impactos positivos “para os enfermeiros”, pois proporcionam a uniformização das práticas assistenciais e a percepção positiva sobre a qualidade dos cuidados prestados, com potencial para ampliar a visibilidade social do papel do enfermeiro nos diversos contextos de trabalho.

*O método* [...] *é importante para uniformizar e prestar melhores cuidados aos doentes.* (E6)

*Nós também temos a nossa expectativa com o nosso local de trabalho* [...] *pretendemos prestar os cuidados com qualidade, fazer o nosso trabalho melhor.* (EE1)

*A metodologia de trabalho que os enfermeiros adotam vai-nos dar visibilidade* [...] *garantir resposta aos melhores cuidados com qualidade e segurança.* (EE5)

Além disso, a implementação de métodos de trabalho adequados tem repercussões na autonomia e satisfação profissional, assim como na estrutura e organização dos cuidados.

*Existem métodos de trabalho que são potenciadores da tomada de decisão e de autonomia* [...] *outros que são francamente mais castradores* [...] *deve-se permitir que o enfermeiro participe ativamente do planeamento, execução e avaliação dos cuidados prestados.* (E7)

*A forma como prestamos e organizamos os cuidados de enfermagem vai influenciar não só a satisfação do cliente, mas também a satisfação dos profissionais no seu local de trabalho, podendo influenciar os indicadores de resultado, aquilo que podemos obter da nossa prática.* (E8)

*A adoção de um método tem uma influência significativa na satisfação dos profissionais. Se os profissionais não estiverem satisfeitos e não estiverem direcionados, o nosso desempenho poderá ficar comprometido, refletindo-se em insatisfação.* (EE5)

Ainda nessa área temática, os depoimentos dos enfermeiros evidenciam efeitos positivos também “para as instituições”, especificamente na eficácia e eficiência organizacionais, promovendo ambientes de prática mais positivos para a enfermagem.

*Se a instituição está direcionada para indicadores de qualidade e se o enfermeiro trabalha para esses indicadores, a forma como organiza o seu trabalho irá refletir-se no aumento de ganhos, satisfação, segurança e qualidade.* (EE4)

*Há métodos de trabalho mais voltados para os indicadores sensíveis aos cuidados de enfermagem* [...] *aqueles que se direcionam para a centralidade no cliente e assim promovem um bom desempenho organizacional.* (EE5)

*O método influencia os ambientes positivos assim como o seu contrário* [...] *o que faz falta muitas vezes à equipa é o chamado salário emocional* [...] *os gestores não conseguem dar uma recompensa monetária* [...] *há reconhecimentos/atitudes que ajudam.* (E7)

## DISCUSSÃO

Independentemente do local onde se desenvolve a assistência de enfermagem aos pacientes, a existência de um método de trabalho adotado pelo enfermeiro é fundamental para obter resultados e projetar a visibilidade do papel social desse profissional.

Desde o início da profissionalização da enfermagem, as questões relacionadas com o trabalho do enfermeiro são discutidas. Já Imogene King alertava para a necessidade de o enfermeiro organizar o seu trabalho. Destacava que a forma como cada profissional assistia os pacientes e a existência de um sistema de documentação dos cuidados permitiriam a distinção da enfermagem das outras profissões da área da saúde, nomeadamente pela identificação das reais necessidades do paciente e familiares cuidadores, bem como pela implementação, avaliação e continuidade dos cuidados^(14-15)^.

No contexto hospitalar, os métodos de trabalho consistem na estrutura do trabalho do enfermeiro, ou seja, a forma pela qual cada enfermeiro, perante o seu desempenho profissional, dá resposta àquilo que é o mandato social da profissão de enfermagem. Percebe-se que o modo como cada profissional de enfermagem identifica as necessidades dos pacientes, formula os diagnósticos, define e implementa as intervenções, assim como avalia os resultados dessas intervenções, possibilita estruturar o trabalho do enfermeiro. Um método de trabalho viabiliza a organização e prestação de cuidados de enfermagem aos pacientes; orienta-se por valores e crenças, sendo descrito como a abordagem independente ou colaborativa dos enfermeiros na prestação de cuidados diretos a um grupo de pacientes^(10,16)^. Importa ainda destacar que a concepção e a organização do trabalho do enfermeiro assentam-se em teorias de administração e essencialmente nas teorias de enfermagem^(10)^, dados corroborados nesta investigação.

Constatou-se que o método de trabalho é central para a definição das intervenções de enfermagem interdependentes e autônomas. O método de trabalho não envolve apenas uma lista de atividades a cumprir, mas também a maneira de os enfermeiros escolherem o que querem fazer, orientados para as reais necessidades do paciente e família^(17)^, com ênfase nas intervenções autônomas de enfermagem. Identificou-se que os métodos de trabalho mais adotados pelos enfermeiros no contexto hospitalar recaíram nos métodos individual, funcional e por enfermeiro de referência, todos voltados para a centralidade do paciente, com implicações positivas na satisfação com os cuidados prestados. Entende se que as metodologias de organização do trabalho do enfermeiro deverão incidir no desenvolvimento do cuidado, atendendo às suas complexidades, assim como o atendimento ao paciente desenvolver-se-á de acordo com as necessidades de atendimento e responsabilidades de cada profissional no ambiente de cuidado^(18)^.

Uma justificativa para o fato de o método de trabalho em equipe não ter sido referido pelos enfermeiros como sendo um método de trabalho em uso foi a concretização do estudo em departamentos, cuja organização dos cuidados de enfermagem se centraliza na pessoa. Além disso, nessa organização, a atribuição dos pacientes aos enfermeiros tem como principal critério a continuidade de cuidados, os quais, sempre que possível, devem ser prestados pelo mesmo enfermeiro.

Foram encontrados alguns facilitadores e dificultadores da implementação dos métodos de trabalho: a escassez de enfermeiros; o dimensionamento de profissionais nos serviços e o número de pacientes atribuídos a cada enfermeiro; as características dos ambientes da prática, nomeadamente a cultura organizacional; o conhecimento detido pelos enfermeiros com reflexo na autonomia na tomada de decisões; e o trabalho em equipe. Autores apontam que a reflexão sobre a prática, o reconhecimento das competências dos enfermeiros e o estilo de liderança são desafios à implementação das metodologias de organização dos cuidados de enfermagem^(5,11,19)^.

Ressalta-se que, além do referido na literatura, observou-se que a complexidade de cuidados, a carga de trabalho, o *turnover* de enfermeiros e pacientes nos serviços, assim como a gestão e liderança no serviço podem influenciar a implementação dos métodos de trabalho. Investigações destacam que a liderança preconizada pelo enfermeiro gestor exerce um papel determinante na qualidade do processo do cuidado e da equipe, porque a forma como um enfermeiro presta cuidados e as competências próprias do gestor tendem a alavancar a qualidade do serviço prestado aos pacientes e família^(6,20-22)^.

Relativamente a outros desafios identificados, os enfermeiros sinalizam para a importância de existir documentos orientadores para a prática assistencial, que permitam uniformizar a prestação de cuidados. Por outro lado, a valorização das competências profissionais e a proatividade dos enfermeiros mostram-se determinantes à implementação dos métodos de trabalho, com vistas à satisfação dos pacientes e profissionais e à qualidade e segurança da assistência de enfermagem.

Ainda percebeu-se que a forma como os enfermeiros organizam o seu trabalho e prestam assistência ao paciente e família terá efeito sobre o paciente, enfermeiros e instituições. A literatura reforça que promover a satisfação dos pacientes, a redução de eventos adversos e de cuidados omissos e uma assistência segura representa ganhos significativos^(6,16)^.

Importa também destacar que a prestação de cuidados de enfermagem sustentada numa metodologia de organização repercute na instituição, pela diminuição dos custos com os cuidados de saúde e, paralelamente, sucesso com a assistência de saúde prestada aos pacientes, num contexto de aumento das despesas com cuidados de saúde, reformas, expectativas e feedback dos consumidores^(14,20,23)^. Evidenciou-se que os métodos de trabalho dos enfermeiros influenciam os ambientes de prática de enfermagem, que se esperam positivos, com a construção de condições de trabalho adequadas, satisfação profissional e com os cuidados prestados e um maior envolvimento dos profissionais com as políticas e estratégias institucionais - achados em linha com a literatura^(24-26)^.

Considerando o vasto conjunto de dados relacionado com a organização do trabalho do enfermeiro^(27)^, torna-se fundamental refletir que, diante dos aspectos discutidos, não há apenas um único método de trabalho capaz de atender às necessidades do paciente e família^(6)^, e sim uma hibridização deles, conforme os fatores que causam essa necessidade. Torna-se premente olhar para esses fatores, nomeadamente, as crenças, valores e percepções dos enfermeiros sobre a sua prática profissional, assim como outros aspectos relacionados com o contexto de cuidados e organizações de saúde, no sentido de garantir, mais eficazmente, a segurança e qualidade em saúde.

### Limitações do estudo

Quanto às limitações, destaca-se o fato de o estudo ter sido realizado somente em alguns departamentos de uma instituição hospitalar do país, o que poderá não traduzir, em sua totalidade, a realidade de outros hospitais de Portugal. Além disso, o número de participantes também pode ser considerado uma limitação, na medida em que pode ter comprometido a identificação de outras perspectivas, em relação à temática em estudo.

### Contribuições para a enfermagem

Considerando as exigências ligadas à qualidade da assistência em saúde, este estudo contribui com as questões relacionadas à organização do trabalho do enfermeiro, na questão da flexibilidade dos enfermeiros para a apropriação do conceito de hibridização de métodos de trabalho alinhados com a cultura organizacional. Os achados mostram-se importantes para a gestão em enfermagem e promoção da qualidade e segurança dos cuidados.

## CONSIDERAÇÕES FINAIS

A organização dos cuidados de enfermagem sustentada em métodos de trabalho apresenta-se como uma premissa necessária para qualidade e segurança dos cuidados. Considera-se que um método de trabalho constitui-se como a estrutura do trabalho do enfermeiro, a qual lhe permite sistematizar a identificação das necessidades, definição dos problemas, implementação das intervenções de enfermagem e avaliação dos cuidados prestados.

Destaca-se que os fatores promotores influenciam a adoção por métodos de trabalho mais direcionados ao cuidado centrado na pessoa, entretanto a presença de desafios reflete-se nas metodologias de organização do trabalho, com potencial impacto nos pacientes, nos enfermeiros e nas instituições, repercutindo na qualidade da assistência. Para isso, sugere-se o estudo dos métodos de trabalho dos enfermeiros que melhor respondem às demandas de cada contexto de cuidados, com sistemático diálogo e acompanhamento dos avanços para qualidade dos cuidados de enfermagem. Importa também sugerir a realização de estudos ligados a essa temática, mas voltados para os enfermeiros gestores, por esses profissionais terem potencial de contribuir significativamente para a gestão do trabalho dos enfermeiros em prol de uma assistência segura e de qualidade aos pacientes.
